# Seasonality of cognitive function in the general population: the Rotterdam Study

**DOI:** 10.1007/s11357-021-00485-0

**Published:** 2021-11-08

**Authors:** Sanne S. Mooldijk, Silvan Licher, Meike W. Vernooij, M. Kamran Ikram, M. Arfan Ikram

**Affiliations:** 1 Department of Epidemiology, Erasmus University Medical Centre, PO Box 2040, 3000 CA Rotterdam, The Netherlands; 2 Department of Radiology and Nuclear Medicine, Erasmus University Medical Centre, Rotterdam, The Netherlands; 3 Department of Neurology, Erasmus University Medical Centre, Rotterdam, The Netherlands

**Keywords:** Brain perfusion, Cerebral blood flow, Cognition, Cosinor, Dementia, Season

## Abstract

Seasonal variation in cognitive function and underlying cerebral hemodynamics in humans has been suggested, but not consistently shown in previous studies. We assessed cognitive function in 10,276 participants from the population-based Rotterdam Study, aged 45 years and older without dementia, at baseline and at subsequent visits between 1999 and 2016. Seasonality of five cognitive test scores and of a summary measure of global cognition were determined, as well as of brain perfusion. Using linkage with medical records, we also examined whether a seasonal variation was present in clinical diagnoses of dementia. We found a seasonal variation of global cognition (0.05 standard deviations [95% confidence interval: 0.02–0.08]), the Stroop reading task, the Purdue Pegboard test, and of the delayed world learning test, with the best performance in summer months. In line with these findings, there were fewer dementia diagnoses of dementia in spring and summer than in winter and fall. We found no seasonal variation in brain perfusion. These findings support seasonality of cognition, albeit not explained by brain perfusion.

**Supplementary Information:**

The online version contains supplementary material available at 10.1007/s11357-021-00485-0.

## Introduction

With aging populations worldwide, it is becoming increasingly important to reliably assess cognitive function and subsequent decline. Beside age-related decline, cognitive function may also show fluctuations over time. Environmental factors might explain such variations in cognitive functioning [1]. For example, ambient temperature and sunlight exposure were shown to influence cognitive functioning [2, 3]. Interestingly, well-known risk factors of cognitive function, such as cardiovascular factors and depressive symptoms, have also been reported to fluctuate throughout the year and may thus mediate these environmental effects [4, 5]. Recognizing such external factors may be relevant in both research and clinical setting to interpret cognitive test scores, to aid further understanding of cognitive functioning, and to guide the development of interventions to improve cognition.

Variation of environmental factors throughout the year, i.e., seasonality, has been studied in relation to cognition [6]. Several studies showed seasonality of some cognitive domains but not of others [7–10], while other studies found no associations at all [11–14]. A study based on data from three community-based cohorts, comprising 3353 participants, found that cognition varied substantially over the year, with higher average global cognitive function around the fall equinox [15]. The seasonal variation was equivalent to an almost 5 years’ difference in age. Moreover, participants examined around the fall equinox were more likely to meet the criteria for mild cognitive impairment or dementia. As was previously noted [6], replication of those results is needed, especially since a peak in cognitive functioning around the fall equinox was found while findings from other studies suggested that performance likely peaks around summer solstice, corresponding to the protective effects of sunlight exposure [3, 16].

Given seasonality of cardiovascular factors, including blood pressure, fluctuations in brain perfusion are a potential underlying mechanisms of cognitive seasonality. Variations in perfusion are associated with performance on cognitive tests and may be influenced by external factors such as ambient temperature [17–19].

Within the population-based Rotterdam Study in the Netherlands, we (1) examined the seasonality pattern of cognition, (2) investigated brain perfusion as a potential underlying mechanism, and (3) assessed whether a seasonality pattern in cognition extends to seasonal variation in the number of clinical dementia diagnoses.

## Methods

### Study setting

The study is embedded within the Rotterdam Study, a population-based cohort study in middle-aged and elderly participants that started in 1990 [20]. The original study population consisted of 7,983 persons aged 55 and older within the Ommoord area, a suburb of Rotterdam. The cohort was expanded by 3,011 persons (≥ 55 years) in 2000 and by 3,932 persons (≥ 45 years) in 2006. Every 3–5 years, participants are re-invited to undergo home interviews and various examinations at the research center. A battery of cognitive tests has been administered during center visits from 1997 onwards, which was further expanded by memory tests from 2002 onwards. Since 2005, brain magnetic resonance imaging (MRI) has been implemented in the Rotterdam Study protocol.

### Study population

From a total of 11,740 participants who attended the research center from 1997 onwards, 10,385 non-demented participants completed at least one cognitive test and were thus eligible for this study. These participants underwent a median of two tests (range: 1–4), with a total of 23,192 cognitive examinations that were available for analysis. Data on brain perfusion was available in a subsample of 5625 non-demented participants whom underwent brain MRI at least once between 2005 and 2016 (11,878 scans). Finally, all participants of the Rotterdam Study who developed dementia during follow-up (*N* = 1970) were included in a separate analysis to assess seasonality of dementia diagnoses.

### Cognitive tests

The initial battery of cognitive tests comprised of the letter-digit substitution test, the verbal fluency test, and the Stroop test. In later examination rounds, the test protocol was further expanded by the Purdue Pegboard test and the 15-word learning test (1999 and 2002, respectively). The Design Organization Test was added between 2009 and 2013 and was not taken into account in the current study because it was only available for a small subset of all cognitive examinations. Details of the cognitive tests have been described previously [21]. Higher scores indicate a better performance on all cognitive tests, except for the Stroop test in which a higher score indicates a worse performance. Scores for the Stroop test were thus inverted for better comparison to other tests.

To obtain a measure for general cognitive function, the g-factor, we performed a principal component analysis incorporating the letter-digit substitution test, the verbal fluency test, the color-word interference subtask of the Stroop test, the delayed recall score of the 15-word learning test, and the Purdue Pegboard test with both hands. Principal component analysis was performed on all 13,654 examinations from 7,883 participants in which all five cognitive tests were completed. The g-factor was identified as the first component of the principal component analysis.

### Brain perfusion

Participants were scanned on a 1.5 T MRI scanner (General Electric Healthcare) [22]. For flow measurement, a two-dimensional phase-contrast imaging was performed as described previously [23]. Briefly, blood flow velocity (in mm per second) was measured using manually drawn regions of interest on the two-dimensional phase-contrast images in both carotids and the basilar artery [24]. For the assessment of brain volumes, the structural MRI scans (T1–weighted, proton density–weighted, and fluid–attenuated inversion recovery) were used [22].

Flow (in mL per second) was calculated by multiplying the average velocity with the cross-sectional area of the vessel. To calculate cerebral blood flow (in mL per minute), flow rates for the carotid arteries and the basilar artery were summer and multiplied by 60 s/min.

We obtained brain perfusion (in mL of blood per minute per 100 mL of brain) by dividing cerebral blood flow by an individual’s brain volume (in mL) and multiplying the result by 100 mL of brain volume. Brain volume was calculated by summing grey and white matter volumes in mL.

### Dementia assessment

Participants were screened for dementia at baseline and follow-up examinations, using a cognitive assessment including the Mini-Mental State Examination (MMSE) [25] and the Geriatric Mental Schedule (GMS) [26] organic level. Participants with an MMSE score lower than 26 or GMS organic level higher than 0 subsequently underwent further investigation and an informant interview including the Cambridge Examination for Mental Disorders in the Elderly (CAMDEX) [27]. A consensus panel led by a consultant neurologist established the final diagnosis dementia according to standard criteria for dementia (Diagnostic and Statistical Manual of Mental Disorders III-revised).

During follow-up, the cohort was under continuous surveillance for dementia incidence through electronic linkage of the database of the Rotterdam Study with medical records from general practitioners and the Regional Institute for Outpatient Mental Health Care. Follow-up for incident dementia is virtually complete until January 1, 2018 (96% of potential person-years).

### Covariables

Participants’ level of education was assessed by interview and was categorized as low (primary education), low/intermediate (lower/intermediate general education or lower vocational education), high general (intermediate vocational or higher general education), or university (higher vocational education or university). Participants were screened for depressive symptoms using the Centre for Epidemiology Studies Depression (CES-D) scale [28] in a home interview which took place before the examinations at the research center.

### Statistical analysis

We compared characteristics of the participants grouped by their season of examination of either cognition or brain perfusion. Seasons were classified according to the meteorological classification, winter (December 1st to February 28/29th), spring (March 1st to May 31st), summer (June 1st to August 31st), and autumn (September 1st to November 30th).

Cognitive test scores were log-transformed in case of a skewed distribution and standardized to allow comparison between the tests. To account for within-person correlations of repeated measurements, we used linear mixed models with random intercepts to analyze seasonality patterns of global cognition and of the individual standardized cognitive test scores. Cerebral blood flow and brain perfusion were analyzed similarly using linear mixed models.

To investigate seasonality patterns, we assessed a sinusoidal pattern with annual seasonality (i.e., *Y(time)* = amplitude * cos(time − horizontal shift), known as a cosinor model. To estimate the amplitude and the horizontal shift, this function was transformed into a regression with sine and cosine terms of the study date (*Y* = *β*_*0*_ + *β*_*1*_* sin(time)* + *β*_*2*_* cos(time)* + *β*_*i*_* X*_*i*_). Dates of study visit were entered as radials (day number in the year / 365.25 * 2π) and back-transformed to dates for presentation of the results. The seasonal variation (amplitude) is the maximal difference between the highest level (peak) and the lowest level (nadir) of the dependent variable throughout the annual period and was calculated as the square root of *β*_*1*_^*2*^ + *β*_*2*_^*2*^.

Detailed descriptions of the estimation of seasonal variations are provided elsewhere [29]. Confidence intervals around seasonal variation were calculated using the delta-method [30, 31]. For visualization of those results for which a statistically significant seasonal variation was found, we also calculated the mean scores for each month by repeating the analyses with terms for the months in the regression instead of the cosinor terms (i.e., *sin(time)* and *cos(time)*).

Because re-examinations of participants of Rotterdam Study cohort waves were scheduled in clustered time periods (e.g., the oldest of the three cohort waves had their fifth re-examination between May 2014 and July 2015), age of participants at time of the examinations is likely to be unbalanced throughout the year. We therefore added terms for cohort wave and age at time of the examination as well as for sex and educational attainment to the models. We additionally adjusted for depressive symptoms (CES-D score) at time of the examination, to explore whether the findings are independent of depressive symptoms, as those are known to vary with the seasons and to also influence cognition [5, 32]. Analyses were performed using examinations with complete covariate data (cognition subset: 22,930 (98.9%) for model 1 and 22,476 (96.9%) for model 2; cerebral blood flow subset: 11,532 (97.1%) for model 1 and 11,416 (96.1%) for model 2).

To test whether the results were driven by a subgroup that is potentially more vulnerable to environmental influences on cognitive functioning, we repeated the analyses in subgroups, namely stratified by age (< 70 and ≥ 70 years), sex, MMSE (< 28 and ≥ 28), after excluding participants who were diagnosed with dementia within 5 years after the study visit, and after excluding participants with a CES-D of 16 or higher, indicative of depressive symptoms. We also repeated the analyses of global cognition after removing individual cognitive tests from the principle component calculation of the global cognition one at a time. Finally, as seasonality of cognition could possibly lead to seasonal variation in dementia, we compared the number of clinical dementia diagnoses per month and per season. For better comparability, the counts were weighted by the number of days in that month or season. We also tested a seasonality pattern using an unadjusted Poisson regression based on the weighted frequencies per month (*ln(count)* = *β*_*1*_* sin(time)* + *β*_*2*_* cos(time)*; with time entered as month in radials) [33].

Analyses were done using R statistical software version 3.6.3 (lme4 package) [34]. Statistical testing was performed two-sided with *P* < 0.05 considered significant. For cosinor models, *P* > 0.05 indicates that no evidence was found that the amplitude of the cosine curve is not equal to zero at any time throughout the year.

## Results

### Study population

Characteristics of the 10,276 participants included in the analyses with cognition (22,930 cognitive tests) and of the 5,445 participants included in the analysis with brain perfusion (11,532 scans) are provided in Table 1 and by season in Supplemental Tables 1 and 2. The average age at cognitive examination was 68.3 [standard deviation (SD) 10.3] and 57% were women. Slightly more participants from the first cohort wave were examined for cognition in winter and fall, but this did not result in large differences in characteristics over the seasons. Of the included 10,276 participants, 7,883 participants (76.7%) completed all five cognitive tests during at least one of their examinations (13,654 examinations; mean age 67.2 [SD 10.9]; 57% women), and thus contribute to the calculation of global cognition. Participants with a cerebral blood flow measurement were younger (mean age 65.4 [SD 9.8]) and less often women (55%).

**Table 1:** Summary characteristics of the study population at time of cognitive examination and of cerebral blood flow measurements

Characteristic	Cognition (*N* = 22,930)^a^	Cerebral blood flow (*N* = 11,532)^a^	*P*-value
Age, years	68.2 (10.3)	65.4 (9.8)	< .001
Women	13,069 (57%)	6328 (55%)	< .001
Caucasian	21,575 (96%)	10,757 (95%)	< .001
Cohort wave			< .001
RS-I	9333 (41%)	1596 (14%)	
RS-II	7279 (32%)	3129 (27%)	
RS-III	6318 (28%)	6807 (59%)	
Education			< .001
Primary	2384 (10%)	877 (8%)	
Lower/intermediate	9509 (41%)	4322 (37%)	
High general	6890 (30%)	3525 (31%)	
University	4147 (18%)	2808 (24%)	
CES-D	8 [1-13]	10 [2-13]	< .001
Depressive symptoms	2805 (12%)	1363 (12%)	.157
Mini Mental State Examination	27.8 (2.0)	28.1 (1.7)	< .001
Cerebral blood flow, mL/min	-	522.3 (100.1)	
Brain perfusion, mL/min per 100 mL	-	55.9 (9.6)	

Values are counts (percentages), means (standard deviation), or median [interquartile range]. *CES-D*, Centre for Epidemiologic Studies Depression scale; *RS*, Rotterdam Study cohort wave. Depressive symptoms are classified as a CES-D score of 16 or higher

^a^Cognitive examinations from 10,276 persons and cerebral blood flow measurements from 5,445 persons

**Table 2:** Seasonality pattern in cognitive tests

Cognitive test	Observations^a^	Mean score (SD)	Seasonal variation (95% CI)	Peak	*P*-value
G-factor	13,654	0.0 (1.0)	0.05 (0.02; 0.08)	Jun. 25th	.001
Letter digit substitution test	22,344	28.2 (7.3)	0.03 (0.01; 0.05)	Mar. 25th	.007
Word fluency test	22,347	21.8 (5.8)	0.01 (− 0.02; 0.04)	Jun. 19th	.366
Stroop					
Reading subtask	21,734	17.8 (4.1)	0.03 (0.00; 0.06)	Jun. 28th	.042
Color naming subtask	21,713	24.5 (6.0)	0.00 (− 0.02; 0.03)	Feb. 1st	.703
Interference subtask	21,642	56.5 (27.6)	0.02 (− 0.01; 0.04)	Mar. 8th	.196
Purdue Pegboard test					
Left hand	17,915	12.2 (2.1)	0.08 (0.04; 0.11)	Jul. 10th	< .001
Right hand	17,777	12.5 (2.1)	0.08 (0.05; 0.12)	Jul. 6th	< .001
Both hands	17,649	10.0 (1.9)	0.10 (0.07; 0.14)	Jul. 16th	< .001
Word learning test					
Immediate	15,169	15.0 (8.1)	0.03 (− 0.01; 0.08)	Apr. 29th	.099
Delayed	15,156	7.2 (3.0)	0.04 (0.00; 0.08)	Jun. 23rd	.040
Recognition	15,261	13.3 (2.1)	0.03 (− 0.01; 0.07)	Sep. 3rd	.160

^a^Number of cognitive tests for which complete data on covariates in model 1 were available. *SD*, standard deviation; *CI*, confidence interval

### Cognition

**Fig. 1:**
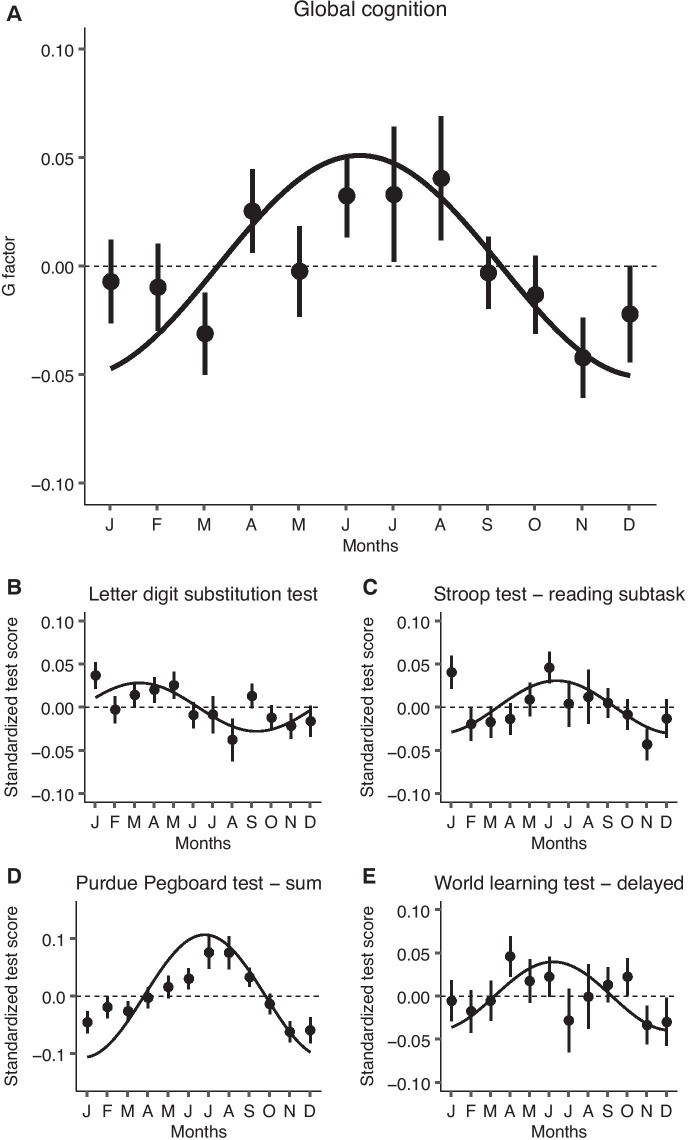
Seasonality of cognition. Seasonal variation of global cognition (**A**) and for standardized individual cognitive tests (**B**–**D**) is plotted as a cosine function (line), based on the amplitude and acrophase (amplitude * cos(t − acrophase)), as estimated in a linear mixed model adjusted for age, sex, cohort wave, and education. The points reflect the adjusted means from a linear mixed model with time included as month, and the corresponding standard errors. The shown individual tests are selected based on indication of a seasonality pattern in the cosinor analysis. For the Purdue Pegboard test, the sum of scores with right, left, and both hands are shown

The g-factor, reflecting global cognitive functioning, explained 52.0% of all variance in the cognitive tests. Global cognition had a seasonal variation with the highest values observed in late June (seasonal variation: 0.05 SD [95% confidence interval (CI): 0.02; 0.08], *P* = 0.001; Table 2; interpretation: cognitive function at time of the peak is 0.05 SD higher than at the nadir). In line with this observation, significantly better performances on the Stroop test (reading subtask), Purdue Pegboard test (left, right, and both hands), and word learning test (delayed task) were observed in summer compared to winter (Table 2). The effect size of seasonal variation ranged from 0.03 SD [95% CI 0.00; 0.06, *P* = 0.042] for the Stroop test (reading subtask) to 0.10 SD [95% CI 0.07; 0.14, *P* < 0.001] for the Purdue Pegboard test with both hands. Figure 1 shows the annual variation of test scores for which a seasonal variation (*P* < 0.05) was found. Similar results were found after additional adjustment for CES-D, in the subgroups, and after excluding individual components from the g-factor calculation one at a time, although not all reached statistical significance (Supplemental Tables 3 and 4).

### Brain perfusion

We found no evidence for a seasonal pattern of cerebral blood flow (seasonal variation: 1.7 mL/min [95% CI − 2.5; 5.9]) or brain perfusion (seasonal variation: 0.3 mL/min per 100 mL brain volume [95% CI − 0.2; 0.7]) when analyzed in the overall study population.

### Dementia diagnoses

**Fig. 2:**
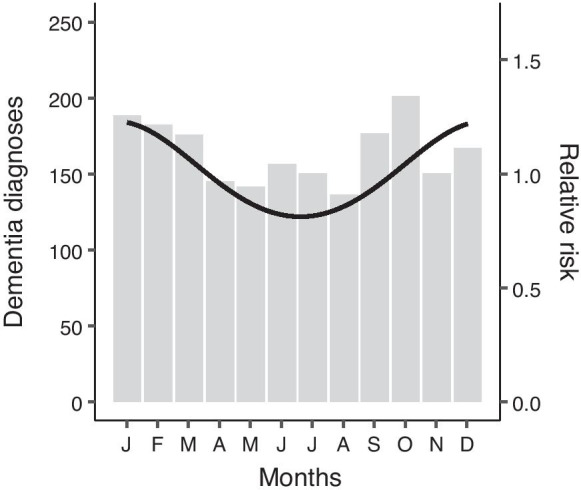
Dementia diagnoses by month. Number of clinical dementia diagnoses by month is presented on the left *y*-axis. Counts are weighted by the number of days in a month to make them comparable. The overlay line, corresponding to the right *y*-axis, shows the result from an unadjusted Poisson regression with cosinor terms

Of all dementia cases in the Rotterdam Study (*N* = 1970), 527 (27.1%; weighted for days per season) persons were diagnosed in winter, 466 (23.5%) in spring, 446 (22.5%) in summer, and 531 (27.1%) in fall. A Poisson regression for the number of dementia diagnoses showed a peak in winter (January) with a seasonal variation of 1.23 [95% CI 1.09–1.40] (interpretation: count at time of the peak is 1.23 times larger than at the nadir). The diagnoses by month, with numbers corrected for the number of days in the season, and the seasonality pattern resulting from the Poisson regression are shown in Fig. 2.

## Discussion

In this study, we found a subtle seasonal variation in cognitive functioning with better performances in summer. Similarly, fewer participants were diagnosed with dementia during summer months. We found no evidence for seasonal variation of brain perfusion as a potential underlying mechanism.

Previous studies on seasonal variation of cognition led to conflicting results, as summarized in a recent review [6]. A study among older adults in the USA, Canada, and France reported a seasonal variation with a peak near the fall equinox, equivalent to an almost 5-year difference in age. Furthermore, they found that during winter and spring, participants were more likely to meet the criteria for mild cognitive impairment or dementia. The authors concluded these findings are of clinical significance and may call for additional dementia care resources in winter and spring. No evidence of a seasonality effect, however, was found in a study of cognitive performance among > 70,000 older adults in New Zealand by Barak et al. [13], and in several other, smaller, studies that predominantly included younger persons [7, 8, 11, 14]. It has been speculated that younger adults might have enough mental resources to overcome the seasonal effects [15] as an explanation for the absence of seasonal variation in the latter studies. Regarding the aforementioned studies of older adults, several methodological differences may explain the contradicting results. First, Lim et al., as well as our study, used cosinor terms in order to analyze the date of examination continuously, whereas Barak et al. compared examinations over the seasons, as 3 months combined (e.g., winter as December–February), implying that cognition does not vary within these categories. Second, Barak et al. analyzed cognition as categories ranging from “intact” to “very severe impairment,” limiting power to assess statistical significance compared to approaches used here such as individual cognitive test scores or a continuous composite measure. Both differences might explain why no seasonality effect was found by Barak et al., especially since a seasonality effect may be subtle, as shown in our current study. Third, latitude of study locations is expected to influence seasonal variation, although this is unlikely to explain the larger seasonality effect on cognition found by Lim et al., as latitudes were similar (52° N in our study versus 41–49° N in the study by Lim et al.).

In the current study, both global cognition and several individual cognitive tests were analyzed. Doing so, we were able to observe that one of the tests in particular, namely the Purdue Pegboard test, reflective of fine motor skills, showed most seasonal variation. A potential explanation for this finding includes a direct influence of ambient temperature, for instance due to cold hands. It should be noted that the seasonality effect of global cognition persisted when the Purdue Pegboard test was not taken into account in the calculation of the g-factor (seasonal variation: 0.04 [95% CI 0.01; 0.06], peak: May 2nd; Supplemental Table 4). Beside ambient temperature, exposure to sunlight is an important season-dependent factor that may drive seasonality effects. An association of sunlight exposure with cognitive functioning has been reported and may have its effect via vitamin D levels, regulations of serotonin and melatonin, and through other mechanisms involved in circadian rhythms [3, 16, 35]. While previous studies found associations of low vitamin D levels with poor cognition [36], it remains to be determined whether the seasonal fluctuations of vitamin D also lead to fluctuations in cognitive functioning. Another potential intermediate of the seasonality effect in cognition is fluctuation of depressive symptoms throughout the year, yet adjustment for CES-D scores, or exclusion of persons with a CES-D ≥ 16 did not largely affect our results (Supplemental Tables 3 and 4). Finally, seasonal variation in dietary habits and particularly of alcohol use may lead to seasonality of cognition. However, this was not supported by findings from a previous study based on Rotterdam Study data in which no seasonal variation in alcohol use was found [37].

Cerebral blood flow has previously been shown to be affected by ambient temperature and by light exposure and is also associated with cognition [17–19, 38]. In this study, we found no evidence for cerebral blood flow or brain perfusion as a neurobiological substrate of seasonality of cognition. This is in line with conclusion from a previous study that the short-term variability of cerebral blood flow is limited [39]. Alternatively, potential seasonal fluctuations in cerebral blood flow may be compensated by cerebral autoregulation, or may be masked by many other factors that influence blood flow.

In accordance with our finding that cognitive performance was highest in summer, the number of dementia diagnoses peaked in winter. Although this could be a consequence of season-related cognitive decline, other factors may also explain the phenomenon. For example, an increase in awareness/recognition of symptoms among family members/caregivers or a decrease in accessibility to medical care during certain times of the year (e.g., holiday season) probably contributes. Nevertheless, results by month suggest that the effect is not solely driven by a reduction in diagnoses in July/August.

Important strengths of this study are the large number of cognition examinations and brain perfusion measurements, and the availability of multiple tests assessing different cognitive domains. Moreover, the thorough ascertainment of dementia cases enabled us to determine whether a seasonality effect also exists in clinical practice. Several limitations also need to be mentioned. First, the five cognitive tests that are required for calculation of global cognition were not completed in all examinations, in part due to a later introduction of the Purdue Pegboard test and the word learning test in the cognitive test battery. However, it does not seem plausible that this relates to seasonality and consequently results in selection bias. Second, although repeated measurements were available, participants were not examined multiple times throughout a year. Such data would allow estimating within-person fluctuations throughout the year and would be valuable to further assess a seasonality effect. Third, linkage of the medical records did not always provide the exact date of diagnosis of the persons that developed dementia during their follow-up. In those cases, a date was assigned or averaged based on the available information (for example, June 15th if the diagnosis was known to be in June).

## Conclusions

In this large population-based study, cognitive functioning had a seasonal variation with slightly better performance in summer, although the effect was subtle compared to the results of a recent cohort study and was mainly visible for tasks testing fine motor skills. In line with these findings, there were fewer dementia diagnoses of dementia in spring and summer than in winter and fall. We found no seasonal variation in brain perfusion. Further research is warranted to determine whether interventions based on environmental factors may have potential to improve cognition.

## Supplementary Information

Below is the link to the electronic supplementary material.

Supplementary file1 (PDF 209 KB)

## Data Availability

Data can be obtained on request. Requests should be directed toward the management team of the Rotterdam Study (secretariat.epi@erasmusmc.nl), which has a protocol for approving data requests. Because of restrictions based on privacy regulations and informed consent of the participants, data cannot be made freely available in a public repository.
