# Supplementary material for: Seasonality of cognitive function in the general population: the Rotterdam Study
Source: GeroScience. 2021 Nov 8;44(1):281–91. doi: 10.1007/s11357-021-00485-0 (PMC8810929; doi:10.1007/s11357-021-00485-0)
Supplement: Supplementary file 1 — Supplementary file1 (PDF 209 KB) [file 11357_2021_485_MOESM1_ESM.pdf]

Seasonality of cognitive function in the general population: the Rotterdam Study

Online Supplemental Material - GeroScience

Sanne S. Mooldijk[1], Silvan Licher[1], Meike W. Vernooij[1,2], M. Kamran Ikram[1,3], M. Arfan Ikram[1*]

[1] Department of Epidemiology, Erasmus University Medical Centre, Rotterdam, the Netherlands

[2] Department of Radiology and Nuclear Medicine, Erasmus University Medical Centre, Rotterdam, The Netherlands

[3] Department of Neurology, Erasmus University Medical Centre, Rotterdam, the Netherlands

*****Corresponding author: M. Arfan Ikram, MD, PhD, Department of Epidemiology, Erasmus University Medical Centre, PO Box 2040, 3000 CA, Rotterdam, The Netherlands. E-mail: m.a.ikram@erasmusmc.nl.

Supplemental Tables

Supplemental Table 1 Characteristics and cognitive test scores of the cognition subset by season

Supplemental Table 2 Characteristics of the brain perfusion subset by season

Supplemental Table 3 Seasonality of cognition adjusted for depressive symptoms

Supplemental Table 4 Seasonality of global cognition in subgroups and after removing individual tests from the calculation of global cognition

Supplemental Table 5 Seasonality pattern of cerebral blood flow and brain perfusion in subgroups

Supplemental Table 1 Characteristics and cognitive test scores of the cognition subset by season

Characteristic	Winter (N=5187)	Spring (N=6536)	Summer (N=3836)	Fall (N=7371)	P-value
Age, years	68.7 (10.0)	67.4 (10.4)	68.5 (10.7)	68.5 (10.3)	<.001
Women	2881 (56)	3738 (57)	2211 (58)	4239 (58)	.111
Caucasian	4910 (96)	6076 (96)	3626 (96)	6963 (96)	.147
Cohort wave					<.001
RS-I	2236 (43)	2613 (40)	1418 (37)	3066 (42)	
RS-II	1725 (33)	2098 (32)	1277 (33)	2179 (30)	
RS-III	1226 (24)	1825 (28)	1141 (30)	2126 (29)	
Education					.005
Primary	480 (9)	670 (10)	385 (10)	849 (12)	
Lower/intermediate	2166 (42)	2687 (41)	1584 (41)	3072 (42)	
High general	1572 (30)	1982 (30)	1144 (30)	2192 (30)	
University	969 (19)	1197 (18)	723 (19)	1258 (17)	
Smoking					.005
Never	1948 (38)	2392 (37)	1338 (35)	2785 (38)	
Former	2442 (47)	3063 (47)	1863 (49)	3330 (46)	
Current	763 (15)	1073 (16)	601 (16)	1175 (16)	
Alcohol use	3413 (81)	4465 (81)	2582 (81)	4722 (81)	.975
BMI, kg/m^2	27.4 (4.2)	27.5 (4.2)	27.3 (4.3)	27.4 (4.3)	.141
Systolic blood pressure, mmHg	145.1 (21.8)	142.2 (21.5)	141.7 (21.9)	143.2 (21.6)	<.001
CES-D	9 [2-13]	7 [1-13]	7 [1-13]	9 [1-13]	<.001
Depressive symptoms	629 (12)	783 (12)	437 (12)	956 (13)	.075
**Cognitive test scores**					
Mini Mental State Examination	27.8 (2.0)	27.8 (2.0)	27.8 (2.0)	27.8 (2.0)	.118
Letter digit substitution test	28.2 (7.2)	28.6 (7.3)	28.0 (7.3)	28.1 (7.3)	<.001
Word fluency test	21.9 (5.8)	21.8 (5.7)	21.8 (6.0)	21.7 (5.7)	.571
Stroop, seconds					
Reading subtask	17.8 (3.9)	17.8 (4.2)	17.8 (4.0)	17.9 (4.0)	.073
Colour naming subtask	24.3 (5.4)	24.4 (6.3)	24.6 (6.1)	24.6 (6.1)	.025
Interference subtask	56.3 (26.7)	55.4 (25.9)	56.9 (29.3)	57.2 (28.7)	.002
Purdue Pegboard test					
Left hand	12.4 (2.2)	12.6 (2.1)	12.5 (2.1)	12.5 (2.2)	<.001
Right hand	12.1 (2.1)	12.2 (2.1)	12.2 (2.0)	12.1 (2.1)	.017
Both hands	9.9 (2.0)	10.0 (2.0)	10.0 (1.9)	10.0 (1.9)	.001
Word learning test					
Immediate	14.9 (8.1)	15.3 (8.3)	15.0 (8.0)	14.9 (8.0)	.042
Delayed	7.1 (3.0)	7.4 (3.0)	7.2 (3.0)	7.2 (2.9)	.001
Recognition	13.3 (2.1)	13.3 (2.1)	13.3 (2.1)	13.3 (2.1)	.657
G-factor	-0.02 (1.02)	0.02 (1.02)	0.03 (1.02)	-0.01 (0.97)	.120

Values are counts (percentages), means (standard deviation) or median [interquartile range]. CES-D, Centre for Epidemiologic Studies Depression scale. Depressive symptoms are classified as a score of 16 or higher.

Supplemental Table 2 Characteristics and cognitive test scores of the brain perfusion subset by season

Characteristic	Winter (N=2852)	Spring (N=3052)	Summer (N=2049)	Fall (N=3579)	P-value
Age, years	65.3 (9.6)	65.4 (10.0)	65.0 (10.2)	65.7 (9.7)	.059
Women	1512 (53)	1684 (55)	1131 (55)	2001 (56)	.126
Caucasian	2650 (95)	2823 (94)	1902 (94)	3382 (95)	.327
Cohort wave					<.001
RS-I	327 (12)	442 (14)	298 (15)	529 (15)	
RS-II	887 (32)	792 (26)	448 (22)	1002 (28)	
RS-III	1638 (56)	1818 (60)	1303 (64)	2048 (57)	
Education					.210
Primary	214 (8)	219 (7)	157 (8)	287 (8)	
Lower/intermediate	1066 (37)	1120 (37)	768 (37)	1368 (38)	
High general	832 (29)	950 (31)	639 (31)	1104 (31)	
University	740 (26)	763 (25)	485 (24)	820 (23)	
Smoking					.003
Never	1097 (39)	1200 (39)	757 (37)	1245 (35)	
Former	1277 (45)	1328 (44)	959 (47)	1677 (47)	
Current	470(16)	513 (17)	328 (16)	644 (18)	
Alcohol use	1933 (83)	1894 (82)	1334 (85)	2539 (84)	.285
BMI, kg/m^2	27.3 (4.1)	27.4 (4.0)	27.4 (4.1)	27.3 (4.0)	.606
Systolic blood pressure, mmHg	139.7 (20.3)	137.8 (20.4)	136.5 (20.1)	138.5 (21.1)	<.001
CES-D	10 [2-13]	10 [3-13]	10 [2-13]	7 [2-12]	<.001
Depressive symptoms	331 (12)	388 (13)	254 (13)	390 (11)	.108
Cerebral blood flow, mL/min	525.8 (102.0)	522.4 (99.2)	523.4 (105.1)	518.9 (96.4)	.047
Brain perfusion, mL/min per 100mL	56.0 (9.7)	55.8 (9.5)	55.9 (9.8)	55.7 (9.4)	.648

Values are counts (percentages), means (standard deviation) or median [interquartile range]. CES-D, Centre for Epidemiologic Studies Depression scale. Depressive symptoms are classified as a score of 16 or higher.

Supplemental Table 3 Seasonality of cognition adjusted for depressive symptoms

Cognitive test	Observations[a]	Mean score (SD)	Seasonal variation (95% CI)	Peak	P-value
G-factor	13,501	0.0 (1.0)	0.05 (0.02; 0.08)	Jun. 19th	.001
Letter digit substitution test	21,915	28.3 (7.2)	0.03 (0.01; 0.05)	Mar. 20st	.002
Word fluency test	21,917	21.8 (5.8)	0.01 (-0.02; 0.04)	Jun. 17th	.385
Stroop					
Reading subtask	21,240	17.8 (4.0)	0.03 (0.00; 0.06)	Jun. 24th	.053
Colour naming subtask	21,307	24.4 (5.7)	0.00 (-0.02; 0.03)	Mar. 24th	.799
Interference subtask	21,240	56.2 (27.5)	0.02 (0.00; 0.05)	Mar. 4th	.059
Purdue Pegboard test					
Left hand	17,704	12.2 (2.1)	0.07 (0.04; 0.11)	Jul. 10th	<.001
Right hand	17,568	12.5 (2.1)	0.08 (0.05; 0.12)	Jul. 6th	<.001
Both hands	17,443	10.0 (1.9)	0.09 (0.06; 0.13)	Jul. 15th	<.001
Word learning test					
Immediate	14,989	15.0 (8.1)	0.04 (0.00; 0.08)	Jun. 4th	.075
Delayed	14,976	7.2 (3.0)	0.04 (0.00; 0.08)	Jun. 22nd	.030
Recognition	15,077	13.3 (2.1)	0.03 (-0.01; 0.07)	Aug. 28th	.161

Results from linear mixed model with cosinor terms, adjusted for age at examination, sex, cohort wave, education and Centre for Epidemiology Studies Depression (CES-D) score (model 2). All cognitive test scores are standardized. Seasonal variation indicates the difference in standard deviation between the estimated lowest and highest scores throughout the year. The peak indicates the date at which the performance for the specific test is expected to be the highest.

The CES-D is reflective of depressive symptoms and higher scores indicate more complaints. CES-D scores peaked in January and had a seasonal variation of 0.56 points (95% confidence interval 0.28-0.84, adjusted using model 1).

[a] Number of cognitive tests for which complete data on covariates in model 1 were available.

SD, standard deviation; CI, confidence interval.

Supplemental Table 4 Seasonality of global cognition in subgroups and after removing individual tests from the calculation of global cognition

Global cognition	Observations[a]	Mean score (SD)	Seasonal variation (95% CI)	Peak	P-value
Total group	13,654	0.00 (1.00)	0.05 (0.02; 0.08)	Jun. 19th	.001
Sex					
Men	5830	-0.10 (0.95)	0.04 (0.00; 0.09)	Jun. 25th	.049
Women	7824	0.08 (1.03)	0.05 (0.02; 0.10)	Jun. 23rd	.005
Age groups					
<70 years	7572	0.45 (0.80)	0.04 (-0.01; 0.09)	Jun. 20th	.100
≥70 years	6082	-0.56 (0.94)	0.02 (-0.02; 0.06)	Jul. 3rd	.371
MMSE					
<28	4285	-0.54 (1.05)	0.05 (-0.01; 0.12)	Aug. 8th	.102
≥28	9320	0.25 (0.87)	0.05 (0.02; 0.08)	Jun. 24th	.004
Dementia <5 years excluded	13,299	0.05 (0.96)	0.05 (0.02; 0.08)	Jun. 21st	.001
CES-D ≥16 excluded	11,707	0.03 (0.99)	0.06 (0.03; 0.09)	Jun. 30th	<.001
Excluding LDST	13,748	0.00 (1.00)	0.06 (0.02; 0.09)	Jul. 10th	.089
Excluding WFT	13,680	0.00 (1.00)	0.05 (0.02; 0.08)	Jun. 17th	.083
Excluding Stroop interference	10,860	0.00 (1.00)	0.06 (0.02; 0.09)	Jun. 21st	.088
Excluding WLT delayed	16,463	0.00 (1.00)	0.04 (0.01; 0.07)	Jun. 24th	.068
Excluding PPB both hands	14,437	0.00 (1.00)	0.04 (0.01; 0.06)	May 2nd	.063

Results from linear mixed model with cosinor terms, adjusted for age at examination, sex, cohort wave and education (model 1). Seasonal variation indicates the difference in standard deviation between the estimated lowest and highest scores throughout the year. The peak indicates the date at which the performance for the specific test is expected to be the highest.
A score of 16 or higher on the Centre for Epidemiology Studies Depression (CES-D) is indicative of depressive symptoms.
[a] Number of cognitive tests for which complete data on covariates in model 1 were available.
SD, standard deviation; CI, confidence interval; LDST, letter digit substitution test; WFT, word fluency test; WLT, word learning test; PPB, Purdue Pegboard test.

Supplemental Table 5 Seasonality pattern of cerebral blood flow and brain perfusion in subgroups

Subgroup	Observations[a]	Mean score (SD)	Seasonal variation (95% CI)	Peak	P-value
**Cerebral blood flow, mL/min**					
Total group	11,878	522.3 (100.1)	1.7 (-2.05; 5.9)	Mar. 9th	.427
Sex					
Men	5204	530.1 (101.5)	1.8 (-4.3; 7.8)	Mar. 13th	.567
Women	6328	516.0 (98.6)	3.4 (-1.9; 8.7)	Feb. 12th	.205
Age groups					
<70 years	7955	543.9 (98.1)	5.7 (0.3; 11.2)	Mar. 26th	.040
≥70 years	3577	476.7 (89.0)	2.0 (-5.4; 9.4)	Jun. 15th	.600
MMSE					
<28	3394	505.5 (99.9)	7.2 (-0.8; 15.2)	Feb. 24th	.078
≥28	8088	529.5 (99.5)	1.2 (-3.8; 6.2)	May 27th	.645
Dementia <5 years excluded	11,341	523.6 (99.8)	1.0 (-3.1; 5.2)	Mar. 4th	.623
**Brain perfusion, mL/min per 100mL**					
Total group	11,878	55.9 (9.6)	0.3 (-00.2; 0.7)	Mar. 18th	.271
Sex					
Men	5204	53.6 (8.9)	0.4 (-0.2; 1.0)	Feb. 17th	.249
Women	6328	57.7 (9.7)	0.4 (-0.2; 1.0)	Feb. 4th	.168
Age groups					
<70 years	7955	56.9 (9.5)	0.6 (0.1; 1.2)	Mar. 27th	.026
≥70 years	3577	53.6 (9.4)	0.1 (-0.8; 0.9)	Jul. 9th	.871
MMSE					
<28	3394	55.2 (9.7)	0.9 (0.1; 1.8)	Mar. 8th	.034
≥28	8088	56.1 (9.5)	0.1 (-0.4; 0.6)	Apr. 12th	.770
Dementia <5 years excluded	11,341	55.9 (9.6)	0.2 (-0.2; 0.7)	Mar. 25th	.354

Results from linear mixed model with cosinor terms, adjusted for age at examination, sex, cohort wave and education (model 1). Seasonal variation indicates the difference in cerebral blood flow or in brain perfusion between the estimated lowest and highest values throughout the year. The peak indicates the date at which the flow or perfusion is expected to be the highest.

[a] Number of scans for which complete data on covariates in model 1 were available.

SD, standard deviation; CI, confidence interval.
